# Phenotypic Profiling of People With Subjective Tinnitus and Without a Clinical Hearing Loss

**DOI:** 10.3389/fncel.2022.804745

**Published:** 2022-02-09

**Authors:** Dongmei Tang, Xiaoling Lu, Ruonan Huang, Huiqian Yu, Wenyan Li

**Affiliations:** ^1^State Key Laboratory of Medical Neurobiology and MOE Frontiers Center for Brain Science, ENT Institute and Department of Otorhinolaryngology, Eye & ENT Hospital, Fudan University, Shanghai, China; ^2^NHC Key Laboratory of Hearing Medicine, Fudan University, Shanghai, China

**Keywords:** tinnitus, normal hearing, audiogram, threshold elevation, high pitch

## Abstract

Our objective was to study the characteristics of patients with subjective tinnitus and normal hearing and to investigate whether the features correlated to different shapes on audiograms. In this retrospective study, 313 patients with subjective tinnitus and clinically normal hearing were enrolled from the tinnitus outpatient department of the Eye and ENT Hospital of Fudan University. The following phenotypic variables were collected: age, dominant tinnitus pitch (TP), tinnitus loudness, tinnitus duration, tinnitus severity, sex, education, hearing thresholds, tinnitus position, and tinnitus condition. The dominant TPs of patients with normal hearing were mostly high-pitched, with a mean of 4866.8 ± 2579.6 Hz; thus, we speculated that the condition is related to high-frequency hearing threshold elevations. We further divided the patients into four subgroups based on the matched TP: (i) TP ≤ 500 Hz (*n* = 34), (ii) 500 Hz < TP ≤ 3,000 Hz (*n* = 15), (iii) 3,000 Hz < TP ≤ 8,000 Hz (*n* = 259), and (iv) TP > 8,000 Hz (*n* = 5). We studied the phenotypic profiling of different audiograms and found that the group with TP of ≤500 Hz had an average “inverted-U” shaped audiogram, and the group with TP between 500 and 3,000 Hz had a slowly ascending slope audiogram below 2,000 Hz, followed by a drastically descending slope audiogram ranging from 2,000 to 8,000 Hz; further, the high-frequency (3,000–8,000 Hz) and ultra-high-frequency (>8,000 Hz) groups had flat curves below 2,000 Hz and steeper slope audiograms over 2,000 Hz. Our findings confirmed a consistency ratio between the distributions of dominant TPs and the frequencies of maximum hearing thresholds in both ears. The dominant TP was positively correlated with the maximum hearing threshold elevation frequency (left ear: *r* = 0.277, *p* < 0.05; right ear: *r* = 0.367, *p* < 0.001). Hearing threshold elevations, especially in high frequency, might explain the appearance of dominant high-frequency TP in patients without clinically defined hearing loss. This is consistent with the causal role of high-frequency coding in the generation of tinnitus.

## Introduction

Tinnitus is the auditory perception of sound in the absence of a corresponding external acoustic or electric stimulus. The commonly described sounds of tinnitus are chirping, buzzing, ringing, and hissing. More often, tinnitus is a tonal-like sensation, but in few cases, it sounds like a noise. Tinnitus affects 5–43% of people worldwide (McCormack et al., 2016). The risk of developing tinnitus increases with age, noise exposure, and hearing loss (Baguley et al., 2013; Langguth et al., 2013). It is widely accepted that the generation of tinnitus is often triggered by peripheral hearing damage and involves a central mechanism (Norena and Eggermont, 2003; Eggermont and Roberts, 2004). In people with hearing loss and tinnitus, the dominant tinnitus pitch (TP) is consistently associated with the region of hearing loss (Konig et al., 2006; Pan et al., 2009; Moore et al., 2010; Sereda et al., 2011, 2015; Schecklmann et al., 2012). However, clinical audiometric hearing loss does not occur in all cases of tinnitus. The percentage of tinnitus patients without clinical hearing loss is estimated at 20% (Jastreboff and Jastreboff, 2003).

One of the most frequently asked questions by patients suffering from tinnitus is “Why did I get tinnitus?,” and the answer is more challenging in the presence of patients with normal hearing. A study assessing the reported lifetime noise exposure found some evidence for cochlear pathology associated with tinnitus and a normal audiogram (≤20 dB HL at 0.25–8 kHz). Guest et al. (2017) conducted a prospective study of 20 people with tinnitus and 20 controls closely matched for sex, age, and audiometric thresholds. Reported lifetime noise exposure was the only factor that differed between groups (Guest et al., 2017). Nevertheless, high-frequency hearing loss also provides some evidence for cochlear pathology associated with tinnitus and a normal audiogram (≤15 dB HL at 0.125 Hz–8 kHz). For example, by conducting a retrospective split of a sample of 75 people with tinnitus, Vielsmeier et al. (2015) found that those with hearing thresholds over 15 dB HL in at least one frequency from 10 to 16 kHz reported higher tinnitus symptom severity as measured by the Tinnitus Questionnaire (TQ) and Tinnitus Handicap Inventory (THI), compared with those with thresholds below 15 dB HL. In our previous study, we reported that extended high-frequency and average hearing thresholds across 4–16 kHz were significantly higher in the tinnitus group than in the matched volunteers without tinnitus (Song et al., 2021). In addition, some researchers have revealed that patients with normal hearing may have missed or hidden hearing loss. One study reported missed hearing loss in patients with normal audiograms when using fine frequency resolution in 1/24 octave step, suggesting that a more sophisticated pure-tone test should be considered to identify mild hearing impairment in patients with normal hearing (Xiong et al., 2019). Another study performed pure-tone audiometry (125 Hz–16 kHz), speech audiometry, transient evoked otoacoustic emissions (TEOAEs), distortion product otoacoustic emissions (DPOAEs), threshold equalizing noise (TEN) test (500 Hz–4 kHz), and electrocochleography (ECochG) for 9 individuals with tinnitus and normal hearing and 13 controls with normal hearing but no tinnitus, and the results showed that the SP/AP ratio in the ECochG test, together with the detection of dead regions in the TEN test, might be valuable in diagnosing hidden hearing loss, while the between-group differences in DPOAE and TEOAE were not significant (Kara et al., 2020).

According to this brief review of the literature, studies on tinnitus patients with normal hearing are mostly limited to detecting abnormalities of cochlear or auditory nerves; however, few studies have attempted to demonstrate the characteristics of pitch, loudness, and severity of tinnitus in patients with normal hearing (Savastano, 2008). Further, few studies have focused on the association between TP and audiogram features in patients with normal hearing. In this study, we focused on phenotypic profile of patients presenting with the primary complaint of subjective tinnitus and clinically normal hearing and confirmed that dominant TP was positively correlated with the maximum hearing threshold elevation frequency, which indicated that a more sophisticated pure-tone test, including extended high frequency, might be needed in evaluating the audiological features of dominant high-frequency TP in patients without clinically defined hearing loss.

## Materials and Methods

### Participants

The study was conducted between January 2014 and October 2017 in tinnitus outpatients of the Eye and ENT Hospital of Fudan University, Shanghai. The recruited patients had a chief complaint of subjective tinnitus. We excluded patients who had pulsatile tinnitus and major health issues that impacted or prevented attendance. Patients with risk factors for tinnitus, such as noise exposure, sudden hearing loss, head or temporal trauma, tympanitis, and emotional disorders, were also identified and excluded. Finally, 313 patients with tinnitus and clinically normal hearing were identified from the database of all tinnitus patients, according to the World Health Organization [WHO] Programme for the Prevention of Deafness and Hearing Impairment (1997) definition of “the pure-tone threshold average across octave frequencies from 0.5 to 4 kHz ≤ 25 dB HL in each ear.” In addition, 71 sex-matched volunteers with normal hearing and without tinnitus were also recruited in this study to compare the audiometric characteristics between the participants with and without tinnitus.

All tests were approved by the ethical committee of Fudan University. The sensitive information relevant to patient privacy was removed before analysis and all participants gave written informed consent to scientific research.

### Audiometry Examination

A pre-exam otoscopic screening was performed to identify abnormalities in the external ear, including the ear canal and eardrum. Excessive or impacted cerumen was immediately cleared. All patients accepted the acoustic immittance measurement, and those with type “C” or type “B” diagram were ruled out. Pure-tone air conduction for both ears was measured between 0.25 and 8 kHz (0.25, 0.5, 1, 2, 4, and 8 kHz). A standard Hughson-Westlake procedure (steps: 10 dB down, 5 dB up; 2 out of 3) was used to determine hearing thresholds.

### Tinnitus Evaluation

A detailed tinnitus evaluation was performed on all participants (see below). We also recorded general information such as age, sex, educational background, self-reported laterality (unilateral, bilateral, or in the head), duration of tinnitus, and sound properties of tinnitus. Both intermittent and continuous tinnitus were included.

### Symptom Severity

Tinnitus severity category was suggested according to the previous report (Biesinger et al., 1998). In brief, Grade I was defined as no impairment, Grade II as a mild complaint with passive emotional impact in certain defined conditions, Grade III as a permanent annoyance due to tinnitus (e.g., insomnia and anxiety), and Grade IV as severe impairment with severe negative impacts on work, study, or daily life.

To comprehensively evaluate the severity of tinnitus, three questionnaires were administered: the THI (Newman et al., 1996, 2008), Fear of Tinnitus Questionnaire (FTQ) (McCracken et al., 1992; Roelofs et al., 2007), and Athens Insomnia Scale (AIS) (Soldatos et al., 2000). The scoring criteria were as follows: (1) THI: slight (0–16), mild (18–36), moderate (38–56), and severe (58–100); (2) AIS: no insomnia (<4), suspicious insomnia (4–6), and insomnia (7–24); and (3) FTQ: 17 items in total, with 1 score per item; the higher the score, the more severe the tinnitus.

### Pitch Matching

We established a “trinomial forced-choice method” for TP matching (China invention patent: ZL 201510165976.9). The first three frequencies were 2, 4, and 8 kHz, which were at least 5 dB above all the corresponding hearing thresholds, and each lasted 500 ms with a 1-s interval. Patients were instructed to identify the frequency closest to their TP and to judge whether it was higher, lower, or equal to their tinnitus. If 2 kHz was selected, then the upper limit frequency of tinnitus was 4 kHz − 1/3 octaves; if 4 kHz was selected, then the frequency of tinnitus was between 2 kHz + 1/3 octaves and 8 kHz − 1/3 octaves; and if 8 kHz was selected, then the lower limit frequency of tinnitus was 8 kHz − 1/3 octaves. In this way, the given sound range was gradually narrowed to the closest frequency by a final step of 1/3 octaves. A threefold repetition was performed to clearly distinguish between each of the three acoustic stimuli. When a final pitch match was selected, an octave confusion test was conducted to avoid selection bias. The octave confusion test conducted in the present study was 1/3 octaves above and 1/3 octaves below the selected pitch (±1 octave when below 1,000 Hz) whenever such frequencies were available. The final confirmed pitch was recorded as the dominant pitch of tinnitus. There were 15 frequencies in total for TP matching, namely, 0.25, 0.5, 1, 1.26, 1.58, 2, 2.52, 3.18, 4, 5.04, 6.35, 8, 10.08, 12.7, and 16 kHz.

### Loudness Matching

The loudness matching was performed in a manner similar to that in the pitch-matching test, and the final gap difference was narrowed to the nearest 1 dB step size. The loudness of tinnitus was reflected in the sensation level (dB SL). Sensation level was defined as the loudness value above the hearing threshold at the TP.

The tinnitus testing devices for pitch and loudness were calibrated twice a year at the Shanghai Institute of Measurement and Testing Technology^1^, which were authorized by the Administration of Quality Supervision, Inspection and Quarantine and accredited by the China National Accreditation Service for Conformity Assessment.

### Data Analysis

The continuous variables are expressed as mean ± standard deviation, while the categorical variables are expressed as number (%). Group comparisons were performed using one-way analysis of variance and Fisher’s exact test with RStudio software (version 4.0.3). Correlation analysis was performed between the dominant TP and corresponding frequency of maximum hearing threshold using the Spearman rank test. All the statistics and plotting were performed with RStudio software (version 4.0.3). The distribution of TP and frequency of maximum hearing threshold was mapped with Excel. The level of statistical significance was defined as a *p*-value of <0.05.

## Results

### Clinical and Demographical Characteristics

According to the WHO grades of hearing impairment (version 1997), 313 subjects conformed to the criteria of clinically normal hearing with standard audiometry. The mean age was 40.2 ± 12.8 years, the male-to-female ratio was 54.3% (*n* = 170): 45.7% (*n* = 143), and nearly half of the patients (47.6%) had a college degree or higher. The mean dominant TP was 4866.8 ± 2579.6 Hz, demonstrating a common dominant high-frequency TP. The pitch-matching analysis showed that most of the matched frequencies (308/313) were no more than 8 kHz, while only five patients reported a dominant TP at 10.08 kHz. To further analyze the data, we divided the participants into four subgroups based on the dominant TP: low-pitched (≤500 Hz), middle-pitched (500–3,000 Hz), high-pitched (3,000–8,000 Hz), and ultra-high-pitched (>8,000 Hz). As shown in Table 1, the four groups were significantly different in terms of age (*p* = 0.037), tinnitus loudness (*p* < 0.001), and sex composition (*p* = 0.025). However, they were homogeneous in terms of duration (*p* = 0.940), severity (*p* = 0.965), position (*p* = 0.334), educational background (*p* = 0.166), and persistence (*p* = 0.089) of tinnitus. The mean duration of tinnitus was 30.7 ± 53.7 months, implying that most patients had chronic tinnitus. There was preponderance of bilateral tinnitus reported by the majority of participants (59.1% in total; 44.1, 66.7, 60.2, and 60.0% in the low, middle, high-, and ultra-high-pitched groups, respectively), with only a few reporting unilateral tinnitus (left 21.1%, right 18.2% in total; left 32.4% vs. right 23.5%, left 6.7% vs. right 20.0%, left 20.5% vs. right 17.8%, and left 20.0% vs. right 20.0% in the low, middle, high-, and ultra-high-pitched groups, respectively). The overall education level of the patients varied; 47.6% had a college degree or higher, 22.0% finished high school or technical school, and 30.4% received primary education or were illiterate. Most patients (85.6%) reported continuous tinnitus, with the remaining patients reporting intermittent tinnitus (Table 1).

**TABLE 1:** Demographic characteristics of patients with tinnitus and clinically normal hearing.

	Total (*n* = 313)	≤500 Hz (*n* = 34)	500–3,000 Hz (*n* = 15)	3,000–8,000 Hz (*n* = 259)	>8,000 Hz (*n* = 5)	*p*-value
**Continuous variable, mean (SD)**						
Age (years)	40.2 (12.8)	40.9 (13.9)	36.0 (15.4)	40.6 (12.4)	25.6 (13.1)	**0.037** *
Dominant tinnitus pitch (Hz)	4866.8 (2579.6)	397.1 (124.9)	1869.3 (484.4)	5526.6 (1978.3)	1,0080 (0)	/
Tinnitus loudness (dB SL)	9.1 (5.2)	10.9 (6.1)	12.1 (9.1)	8.5 (4.6)	16.0 (6.5)	**<0.001** *
Tinnitus duration (months)	30.7 (53.7)	25.8 (38.4)	32.3 (37.7)	31.4 (56.7)	24.4 (23.5)	0.940
THI score	31.0 (23.3)	30.5 (19.8)	35.1 (27.0)	31.0 (23.6)	21.2 (17.5)	0.716
AIS score	5.9 (5.3)	5.8 (5.7)	7.3 (4.7)	5.8 (5.3)	3 (3.7)	0.454
FTQ score	8.1 (4.0)	7.9 (3.5)	9.7 (4.1)	8.0 (4.0)	6.4 (4.3)	0.306
**Categorical variable, n (%)**						
**Sex**						
Male	170 (54.3%)	11 (32.4%)	7 (46.7%)	150 (57.9%)	3 (60.0%)	**0.025** *
Female	143 (45.7%)	23 (67.6%)	8 (53.3%)	109 (42.1%)	2 (40.0%)	
**Education**						
Below high school	95 (30.4%)	15 (44.1%)	2 (13.3%)	78 (30.1%)	0 (0)	
High school or technical school	69 (22.0%)	8 (23.5%)	4 (26.7%)	55 (21.2%)	2 (40.0%)	0.166
College degree or above	149 (47.6%)	11 (32.4%)	9 (60.0%)	126 (48.7%)	3 (60.0%)	
**Tinnitus position**						
Left ear	66 (21.1%)	11 (32.4%)	1 (6.7%)	53 (20.5%)	1 (20.0%)	
Right ear	57 (18.2%)	8 (23.5%)	3 (20.0%)	46 (17.8%)	1 (20.0%)	0.334
Bilateral ears	185 (59.1%)	15 (44.1%)	10 (66.7%)	156 (60.2%)	3 (60.0%)	
In head	5 (1.6%)	0 (0)	1 (6.6%)	4 (1.5%)	0 (0)	
**Tinnitus severity**						
Grade I	5 (1.6%)	1 (2.9%)	0 (0)	4 (1.5%)	0 (0)	
Grade II	124 (39.6%)	12 (35.3%)	6 (40.0%)	103 (39.8%)	3 (60.0%)	0.965
Grade III	140 (44.7%)	17 (50.0%)	7 (46.7%)	114 (44.0%)	2 (40.0%)	
Grade IV	44 (14.1%)	4 (11.7%)	2 (13.3%)	38 (14.7%)	0 (0)	
**Tinnitus condition**						
Intermittent	45 (14.4%)	8 (23.5%)	4 (26.7%)	32 (12.4%)	1 (20.0%)	0.089
Continuous	268 (85.6%)	26 (76.5%)	11 (73.3%)	227 (87.6%)	4 (80.0%)	

*The bold values represent the variable items compared between the four subgroups. n means number and SD means standard deviation.*

*P < 0.05.

### Tinnitus Loudness and Severity

The mean tinnitus loudness was 9.1 ± 5.2 dB SL above the threshold for all patients with tinnitus and normal hearing. To be visualized, the level of loudness was mid-range; hence, the tinnitus was hardly masked by the surrounding ambient sounds, causing disruption to patients’ daily lives, sleep, and work. The mean loudness measured by sensation level was 10.9 ± 6.1, 12.1 ± 9.1, 8.5 ± 4.6, and 16.0 ± 6.5 in the low, middle, high-, and ultra-high-pitched groups, respectively (*p* < 0.001). According to the Biesinger classification, 1.6% of patients had no impairment (Grade I), 39.6% had slight impairment (Grade II), 44.7% had permanent annoyance (Grade III), and 14.1% had severe impairment (Grade IV), with no differences between the four subgroups. This implied that most patients with tinnitus and normal hearing were slightly or severely disturbed in private and professional areas. These findings were reconfirmed by the three questionnaire assessments: the THI global score was 31.0 ± 23.3, AIS score was 5.9 ± 5.3, and FTQ score was 8.1 ± 4.0 (Table 1).

### Dominant Tinnitus Pitch of Clinically Normal Hearing Might Be Associated With Hearing Threshold Elevations

In previous studies, TP has been found to be consistent with the region of hearing loss (Sereda et al., 2011, 2015; Schecklmann et al., 2012). However, the cause of tinnitus without a clinical hearing loss remains unclear, especially for those who do not have any hearing deficits at all test frequencies ranging from 250 to 8,000 Hz. Among all the features of tinnitus with normal hearing, the average high-pitched-matching result was the most salient and intriguing. We speculated that the generation of tinnitus and the dominant TP in patients with normal hearing might be related to different audiogram types. We drew the average pure-tone audiograms for each ear in the low, middle, high-, and ultra-high-pitched subgroups. As a contrast, 71 volunteers with normal hearing and without tinnitus also received the standard pure-tone audiometry in the same audiometric environment with the patients with tinnitus.

**FIGURE 1:**
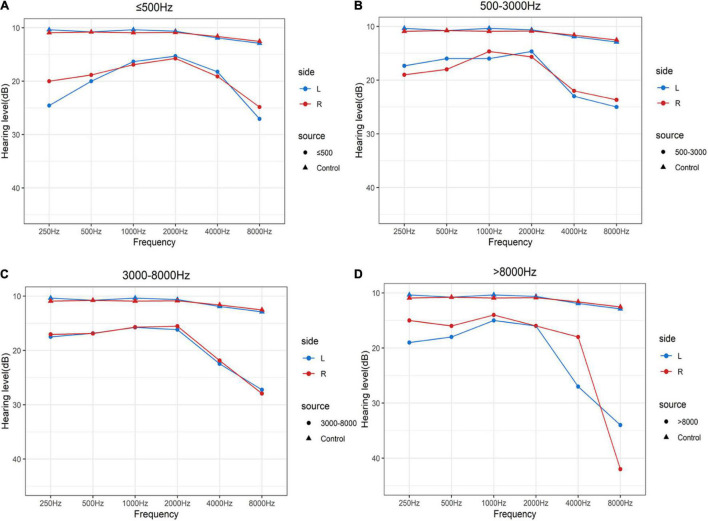
Average pure-tone audiograms of patients with clinically normal hearing and volunteers without tinnitus using a 250–8,000 Hz conventional audiometer. **(A–D)** Mean values of average hearing thresholds for the controls with normal hearing and without tinnitus and patients with clinically normal hearing in dominant tinnitus pitch ≤500 Hz **(A)**, 500 Hz–3 kHz **(B)**, 3–8 kHz **(C)**, and >8,000 Hz **(D)**. Blue solid lines and dots represent left ears and red solid lines and dots represent right ears in patients with tinnitus. Blue solid lines and triangles represent left ears and red solid lines and triangles represent right ears in volunteers with normal hearing and without tinnitus.

As shown in Figure 1 and Table 2, the control group totally had flat audiograms both in right ears and left ears, and the average pure-tone thresholds were all less than those in patients with tinnitus from 250 to 8,000 Hz. To clearly compare the audiogram curves between tinnitus patients and the controls, the average pure-tone audiogram of volunteers without tinnitus was presented repeatedly in the four subgroups. The average pure-tone audiograms in the low-pitched (≤500 Hz) group presented an inverted-U shape in both left and right ears, indicating that dominant low-pitched tinnitus may be associated with hearing threshold elevations ranging from 250 to 500 Hz (Figure 1A). The inclination and intensity of threshold elevations in low frequencies were attenuated in the middle-pitched (500–3,000 Hz) subgroup when compared to the low-pitched subgroup, demonstrating a slowly ascending slope audiogram below 2,000 Hz, followed by a drastically descending slope audiogram ranging from 2,000 to 8,000 Hz (Figure 1B). In the high- (3,000–8,000 Hz) and ultra-high- (above 8,000 Hz) pitched subgroups, both the audiogram curves below 2,000 Hz were flat without any severe threshold elevations, while the audiograms over 2,000 Hz revealed steeper slopes in both ears (Figures 1C,D). Therefore, widespread threshold elevations in high frequencies (over 2,000 Hz) were found in all the four subgroups, which might contribute to reasonable explanation for our observation that the majority of patients with normal hearing had a dominant high-frequency TP. However, the threshold elevations in low frequencies (250–500 Hz) and the elevated degrees would control the final distribution of TP. The inverted-U-shaped audiogram in patients with normal hearing might indicate a low-pitched tinnitus.

**TABLE 2:** The average pure tone thresholds (mean) and the corresponding standard deviations (SD) in different subgroups with tinnitus and the control group without tinnitus.

Frequency		Hearing thresholds [mean (SD), dB HL]				
		Control (*n* = 71)	≤500 Hz (*n* = 34)	500–3,000 Hz (*n* = 15)	3,000–8,000 Hz (*n* = 259)	>8,000 Hz (*n* = 5)
250 Hz	L	10.4 (4.7)	24.6 (18.4)	17.3 (3.7)	17.5 (5.1)	19.0 (7.4)
	R	10.9 (4.3)	20.0 (5.9)	19.0 (3.4)	17.0 (4.9)	15.0 (5.0)
500 Hz	L	10.8 (5.0)	20.0 (7.4)	16.0 (3.4)	16.9 (4.4)	18.0 (5.7)
	R	10.8 (3.9)	18.8 (4.8)	18.0 (3.7)	16.9 (4.3)	16.0 (4.2)
1,000 Hz	L	10.4 (3.9)	16.3 (4.3)	16.0 (7.1)	15.8 (4.1)	15.0 (3.5)
	R	10.9 (3.9)	16.9 (4.4)	14.7 (3.0)	15.7 (3.8)	14.0 (4.2)
2,000 Hz	L	10.6 (3.3)	15.3 (4.8)	14.7 (4.4)	16.2 (5.1)	16.0 (5.5)
	R	10.8 (3.4)	15.7 (5.5)	15.7 (3.7)	15.5 (4.5)	16.0 (6.5)
4,000 Hz	L	11.9 (5.4)	18.2 (6.4)	23.0 (11.9)	22.5 (10.0)	27.0 (12.5)
	R	11.6 (4.1)	19.1 (8.4)	22.0 (12.6)	21.9 (9.9)	18.0 (5.7)
8,000 Hz	L	12.9 (4.6)	27.1 (16.7)	25.0 (13.0)	27.2 (14.1)	34.0 (16.4)
	R	12.5 (4.6)	24.9 (14.0)	23.7 (11.1)	27.9 (14.9)	42.0 (29.7)

**FIGURE 2:**
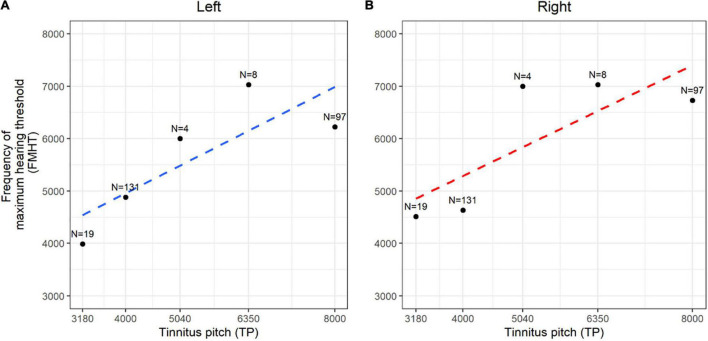
Spearman rank correlation between dominant tinnitus pitch (TP) and frequency of maximum hearing threshold (FMHT). **(A,B)** The correlation coefficient and generalized linear regression between the matched tinnitus pitch and the frequency of maximum hearing threshold were demonstrated in the left ears (**A**, blue dotted line) and right ears (**B**, red dotted line). N means the counts of each data.

We further examined the association between TP and the frequencies of hearing threshold elevation and found that TP was correlated to the frequency of maximum hearing threshold in both ears, with a coefficient of association of 0.277 in the left ears (*p* = 0.015) (Figure 2A) and 0.367 in the right ears (*p* < 0.001) in the high-pitched subgroup (Figure 2B). This finding confirmed that the dominant TP might be speculated by the different audiograms of patients, indicating that minor pure-tone threshold elevation ranging from 250 to 8,000 Hz might be responsible for the generation of tinnitus in individuals with normal hearing.

**FIGURE 3:**
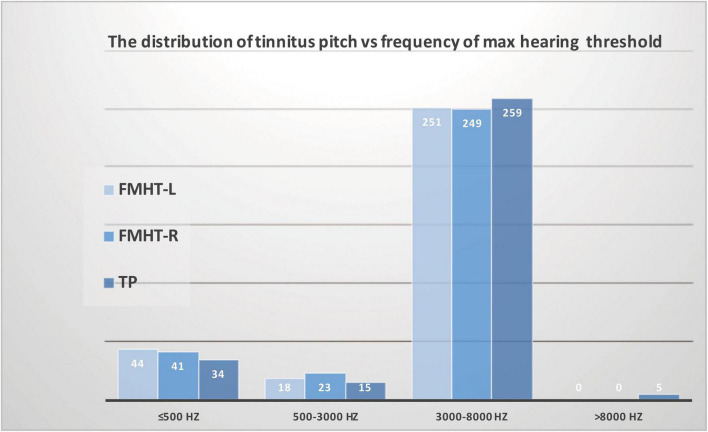
Association between the distribution of tinnitus pitch and the distribution of maximum threshold corresponding frequency in patients with tinnitus and normal hearing. The distribution of threshold-elevated frequencies in both the left ears and right ears was consistent with the distribution of dominant tinnitus pitch.

Moreover, we analyzed the distribution of dominant TP vs. the distribution of maximum threshold-elevating frequencies in both ears and found that the distributions of threshold-elevating frequencies in both ears were consistent with the distribution of dominant TP. Furthermore, of the 313 participants, 259 had high-pitched tinnitus, 251 had maximum hearing threshold in the high frequency range (3,000–8,000 Hz) in their left ears, and 249 had maximum hearing threshold in the high frequency range (3000–8000 Hz) in their right ears. These results confirmed our explanation that the matched dominant TP was mostly high-pitched in patients without clinical hearing loss (Figure 3). Our findings provide noteworthy links between tinnitus pitch and subclinical threshold elevations in the audiometric profile within the testing range of conventional audiometer at the first time, indicating a causal role of mild threshold elevation in the generation of tinnitus. Additionally, the pitch of tinnitus might be predicted from different types of audiograms.

## Discussion

It is well-known that the presence of tinnitus is strongly associated with hearing loss, and that the dominant TP is often within the region of hearing loss. However, tinnitus is not limited to individuals with a clinically measurable hearing loss. To date, only a few studies have examined the association between tinnitus and “subclinical” threshold elevations in the audiometric profile. Previous research mainly focused on discovering the existence of ultra- or extended high-frequency (above 8 kHz) hearing loss in patients with tinnitus and normal hearing, as well as the age-, sex-, and audiometry-matched controls without tinnitus; however, they failed to find a close relationship between TP and the frequency of maximum hearing loss or edge frequency. The possible reason might be partially attributed to the absence of TP-matching procedure in extended high-frequency regions. In the present study, we performed the “trinomial forced-choice method” for tinnitus matching, with 16 frequency measurement points ranging from 250 Hz to 16 kHz. Nevertheless, 308 of the 313 participants reported matched TPs no higher than 8 kHz, indicating that ultra-high frequency hearing loss might be insufficient evidence for the generation of tinnitus in patients with normal hearing and that the profiling of conventional audiometry (125 Hz–8 kHz) data should be investigated further. Hence, this study sought to verify whether minor audiometric deficits up to 8 kHz play a defining role in the phenotypic profiling of tinnitus in patients with normal hearing.

Our results found that the dominant TP of patients with clinically normal hearing was almost within the high-frequency range (especially at 4 and 8 kHz), with a mean of 4866.8 ± 2579.6 Hz. In several studies, Paglialonga et al. (2010, 2011) have established that normal hearing (with mean thresholds ≤ 20 dB HL at 0.5–4 kHz and ≤ 40 dB at 8 kHz) in patients with tinnitus does not preclude a completely normal hearing level for someone with cochlear dead regions or outer hair cell damage, particularly at high frequencies. This pattern is frequently observed when compared to controls (Paglialonga et al., 2010, 2011). In the present study, we demonstrated a more direct correspondence between TPs and the frequencies of hearing deficits according to the audiometric profiles. The participants were divided into four groups based on the pitch-matching results: low-frequency (≤500 Hz), middle-frequency (500–3,000 Hz), high-frequency (3,000–8,000 Hz), and ultra-high-frequency (>8,000 Hz). Interestingly, 82.7% of the participants had high-pitched tinnitus in the 3–8 kHz range, with audiograms characterized by dramatical threshold elevations above 2 kHz in a sharp descending slope and flat curve below 2 kHz with no obvious threshold elevations. A similar phenotypic profiling of audiograms was examined in patients with ultra-high-pitched tinnitus. Approximately 10.9% of the individuals presented low-pitched tinnitus with distinctive inverted-U-shaped audiograms in both ears, suggesting comparable threshold elevations in the low-frequency region (≤500 Hz) vs. the high-frequency region (4–8 kHz). Furthermore, we confirmed a positive correlation between TP and frequency of maximum hearing threshold in both right and left ears. Additionally, the distribution of TP was proven to be consistent with the distribution of maximum hearing threshold corresponding frequency. Therefore, our results indicated that minor hearing deficits might be the cause of tinnitus in patients with normal hearing and that the TP might be predicted by different audiograms.

Tinnitus severity and subjective tinnitus-related discomfort are often more severe in subjects with hearing loss than in those with normal hearing. However, Yenigun et al. (2014) found that the tinnitus severity index and THI results are significantly similar between the groups with normal hearing and hearing loss. Our data did not show a significant between-group difference in THI score when the patients were divided into subgroups based on the TP (*p* = 0.716). The mean score for THI was 31.0 ± 23.3, and according to the scoring criterion for THI, the majority of patients with tinnitus and normal hearing experienced mild to moderate disturbance. The assessment of tinnitus also requires multi-dimensional psychoacoustic evaluation. Furthermore, we used AIS and FTQ to evaluate sleep disorders and fear of tinnitus in patients with normal hearing, and the results were similar to those obtained with THI, with no significant between-group difference (*p* = 0.454 for AIS and *p* = 0.306 for FTQ).

In summary, we expound the characteristics of tinnitus in patients with clinically normal hearing in terms of age, sex, education, hearing threshold, tinnitus duration, tinnitus severity, dominant TP, laterality, tinnitus loudness, multi-dimensional evaluation scales, and intermittent vs. continuous tinnitus. We discovered that the dominant TP was mostly in the high-frequency range, and that the dominant TP in patients with tinnitus and normal hearing might be related to threshold elevations up to 8 kHz. On dividing the patients into four subgroups based on the TP, we identified different types and distinctive audiograms in the low-pitched (≤500 Hz), middle-pitched (500 Hz–3 kHz), high-pitched (3–8 kHz), and ultra-high-pitched subgroups. Therefore, our results suggest that the details of conventional audiogram should be well-studied in patients with tinnitus and normal hearing. We believe that our results at least partially explicit the common question “Why did I get tinnitus?” in patients with normal hearing from the aspect of audiogram. The efficacy of tinnitus management involving different subgroups in patients with normal hearing is worthy to study in the future.

## Data Availability Statement

The raw data supporting the conclusions of this article will be made available by the authors, without undue reservation.

## Ethics Statement

The studies involving human participants were reviewed and approved by the Institutional Review Board (IRB) of Eye & ENT Hospital of Fudan University. Written informed consent to participate in this study was provided by the participants’ legal guardian/next of kin.

## Author Contributions

DT and XL performed the experiments and drafted the manuscript. DT, RH, and HY analyzed the data and approved the final manuscript. DT and WL conceived and designed the study, interpreted and analyzed the data, and approved the final manuscript. All authors contributed to the article and approved the submitted version.

## Conflict of Interest

The authors declare that the research was conducted in the absence of any commercial or financial relationships that could be construed as a potential conflict of interest.

## Publisher’s Note

All claims expressed in this article are solely those of the authors and do not necessarily represent those of their affiliated organizations, or those of the publisher, the editors and the reviewers. Any product that may be evaluated in this article, or claim that may be made by its manufacturer, is not guaranteed or endorsed by the publisher.

## Footnotes

1
http://english.simt.com.cn
